# Case Report: Efficacy of preoperative conversion therapy with lenvatinib, toripalimab, and hepatic arterial infusion chemotherapy in an advanced hepatocellular carcinoma

**DOI:** 10.3389/fonc.2025.1627281

**Published:** 2025-10-08

**Authors:** Jing Mao, Hengzhi Zhang, Xingxia Yang, Yanjun Yao, Xu Sun, Qiang Yan

**Affiliations:** Huzhou Central Hospital, Affiliated Huzhou Hospital, Zhejiang University School of Medicine, Huzhou, Zhejiang, China

**Keywords:** lenvatinib, toripalimab, hepatic arterial infusion chemotherapy, hepatocellular carcinoma, case report

## Abstract

Advanced hepatocellular carcinoma (HCC) has a poor prognosis. Conversion therapy based on non-surgical local therapy and drug therapy is an effective treatment for unresectable HCC. We report a case of HCC with intrahepatic metastasis and multiple venous tumor thrombi. After nearly 4 months of conversion therapy with lenvatinib, toripalimab combined with hepatic arterial infusion chemotherapy (HAIC), the lesions were significantly reduced, the portal vein tumor thrombus resolved, and the alpha-fetoprotein (AFP) decreased to the normal range. This patient had no serious adverse events during treatment. After a comprehensive assessment of the patient’s status, the patient eventually underwent curative surgical resection of the tumor and had a complete response. The successful experience of this case indicates that lenvatinib, toripalimab combined with HAIC has a good prospect as a conversion therapy for HCC with intrahepatic metastasis and multiple venous tumor thrombi.

## 1 Introduction

Hepatocellular carcinoma (HCC) is a common malignant tumor. The early stage of HCC lacks typical clinical symptoms, and most of the diagnosed patients are in the middle and late stages. The prognosis of these patients is often poor. Global cancer data in 2022 from 185 countries showed that 865,269 new cases of liver cancer and 757,948 new deaths were caused by liver cancer in the whole year (1). HCC is the main type of liver cancer. Portal vein tumor thrombus formation (PVTT) is a common complication of HCC, which occurs in about 10%-40% of HCC patients (2). Once combined with PVTT, HCC patients will quickly develop complications such as intrahepatic and extrahepatic metastasis, ascites, jaundice, portal hypertension, and so on. The median survival time of untreated patients is only 2.7-4.0 months (3). The incidence of HCC with inferior vena cava tumor thrombus (IVCTT) is lower than that of PVTT. However, the prognosis of these patients is extremely poor, and most of them will develop liver failure in a short time or pulmonary embolism, pulmonary dissemination, heart failure and even sudden death after tumor thrombus detachment. Without timely treatment, the median survival time is only 2–4 months (4).

Barcelona Clinic Liver Cancer Staging (BCLC) classifies liver cancer with PVTT/IVCTT as advanced stage. Molecular targeted drugs such as sorafenib and lenvatinib are recommended as first-line treatment for patients in this stage (5). Lenvatinib, an oral multi-targeted tyrosine kinase inhibitor, has been recommended as a first-line treatment for unresectable HCC by the U.S. Food and Drug Administration since its non-inferiority to sorafenib in the treatment of HCC was confirmed (6, 7). Toripalimab is a recombinant humanized programmed death receptor-1 (PD-1) monoclonal antibody, which has been approved by the National Medical Products Administration for the treatment of unresectable or metastatic melanoma after failure of previous systemic therapy. It is the first domestic PD-1 inhibitor. Toripalimab has also been shown to be effective in the treatment of HCC. However, even with tyrosine kinase inhibitors combined with immunotherapy, the median survival time of patients with BCLC stage C HCC is generally less than one year (8).

Hepatic arterial infusion chemotherapy (HAIC) involves the delivery of chemotherapy drugs directly into the hepatic artery via peripheral vessels to kill tumor cells. Compared with systemic chemotherapy, HAIC allows high-dose drug delivery to the liver tumors while reducing systemic exposure due to the first-pass effect, thereby enhancing antitumor efficacy and minimizing toxicity. The therapeutic effect of HAIC in HCC patients with PVTT has also been verified. A prospective randomized controlled study showed that HAIC treatment group could achieve much higher objective response rate and median survival time than sorafenib treatment group (9). Therefore, HAIC has been recommended for patients with unresectable HCC with PVTT in the Chinese expert consensus guidelines (10). In addition, A study in Japan also suggested the use of HAIC in advanced HCC to improve the prognosis of patients (11).

For patients with extensive invasion of tumor or tumor thrombus, the use of targeted therapy, immunotherapy, HAIC and other treatments can often reduce the scope of tumor or tumor thrombus and the number of satellite lesions, so that patients can achieve the purpose of radical resection, which is called conversion therapy. However, there is no definitive treatment protocol for conversion therapy of unresectable advanced HCC. Here, we report a patient with HCC intrahepatic metastasis with portal vein and inferior vena cava tumor thrombus who achieved radical resection of HCC after conversion therapy with lenvatinib, toripalimab combined with HAIC.

## 2 Case description

**Figure 1:**
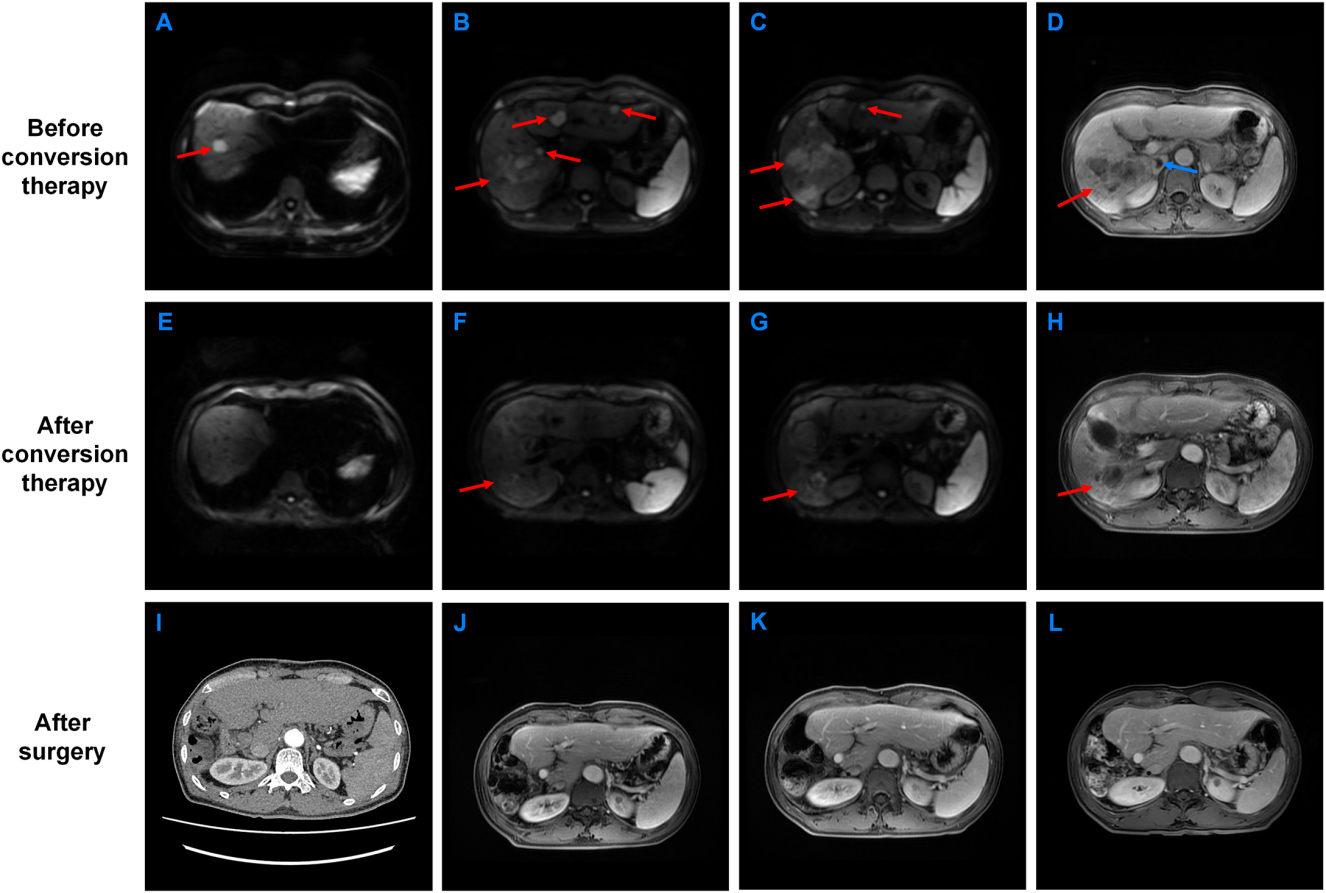
Abdominal magnetic resonance imaging (MRI) and computed tomography (CT) of the patient before and after treatment. **(A–C)** MRI (DWI) of the patient before conversion therapy. **(D)** MRI (venous phase) of the patient before conversion therapy. **(E–G)** MRI (DWI) of the patient after conversion therapy. **(H)** MRI (venous phase) of the patient after conversion therapy. **(I)** The patient’s CT at 1 month after surgery. **(J)** The patient’s MRI at 1 year after surgery. **(K)** The patient’s MRI at 2 years after surgery. **(L)** The patient’s MRI at the most recent follow-up. Red arrows represent tumor lesions and blue arrows represent venous tumor embolus.

**Figure 2:**
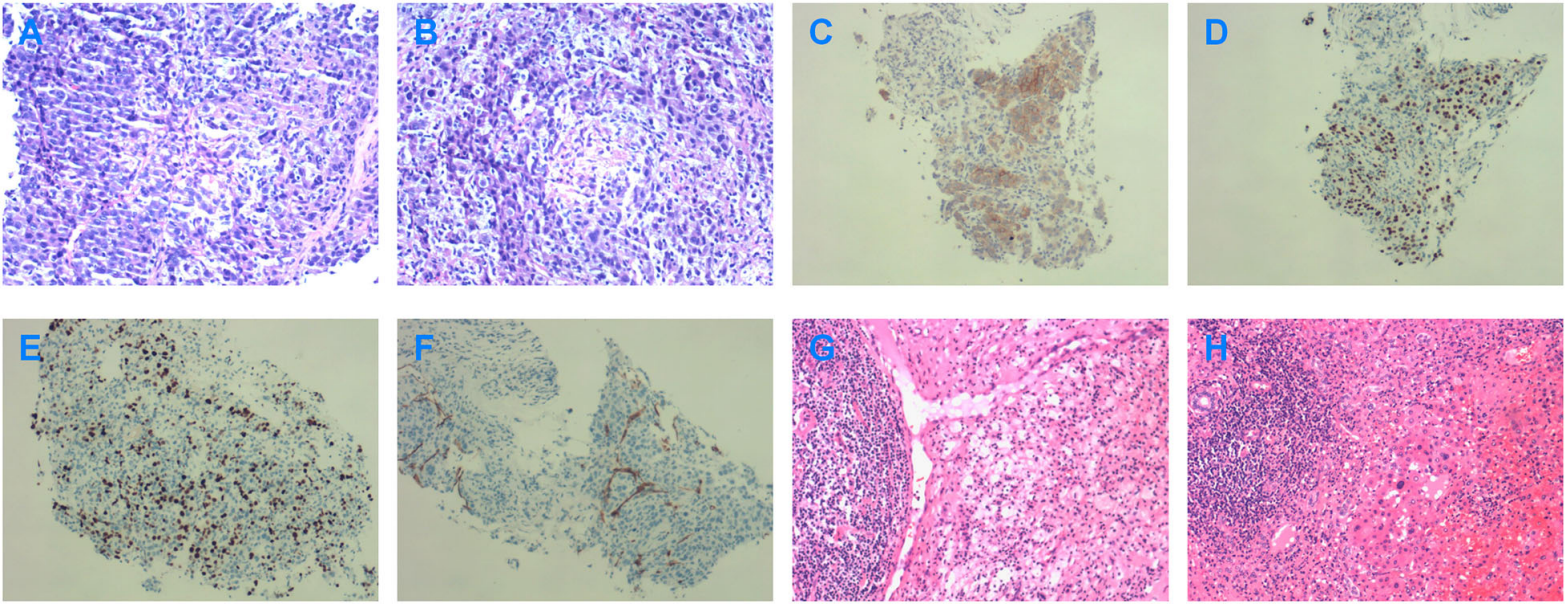
Hematoxylin-eosin (HE) staining and immunohistochemistry (IHC) of tumor samples from the patient. **(A, B)** HE staining of puncture biopsy samples from the patient. **(C–F)** IHC (GPC-3, AFP, Ki-67 and CD34) of puncture biopsy samples from the patient. **(G, H)** HE staining of surgically resected tumor samples from the patient.

The patient was a 59-year-old man with a history of viral hepatitis B for more than 20 years. On May 11, 2021, he came to hospital for upper abdominal enhanced magnetic resonance imaging (MRI) due to discomfort of the upper abdomen. He was found to have multiple intrahepatic masses, including a large mass measuring approximately 132×89×57 mm in the right lobe of the liver with partial thrombosis of the right branch of the portal vein and thrombosis of the inferior vena cava (**Figures 1A–D**). The alpha-fetoprotein (AFP) was exceeded 200,000 ng/mL. Therefore, a diagnosis of liver malignancy with multiple intrahepatic metastases was made. On admission, the patient was conscious. There was no jaundice of the skin and sclera, a flat and soft abdomen, no tenderness or rebound pain, and a Child-Pugh A liver function. This patient has underlying liver cirrhosis, but he denies having a family history of liver cancer. Based on the patient’s medical history and examination results, HCC was strongly suspected. However, differential diagnoses such as intrahepatic cholangiocarcinoma, mixed liver cancer, and metastasis from an extrarenal primary tumor could not be ruled out either. Therefore, the patient underwent ultrasound-guided needle biopsy of the lesion. This report suggests HCC (**Figures 2A, B**). Immunohistochemical results showed that GPC-3, AFP, Ki-67 and CD34 were positive (**Figures 2C–F**). This further clarifies the diagnosis.

**Figure 3:**
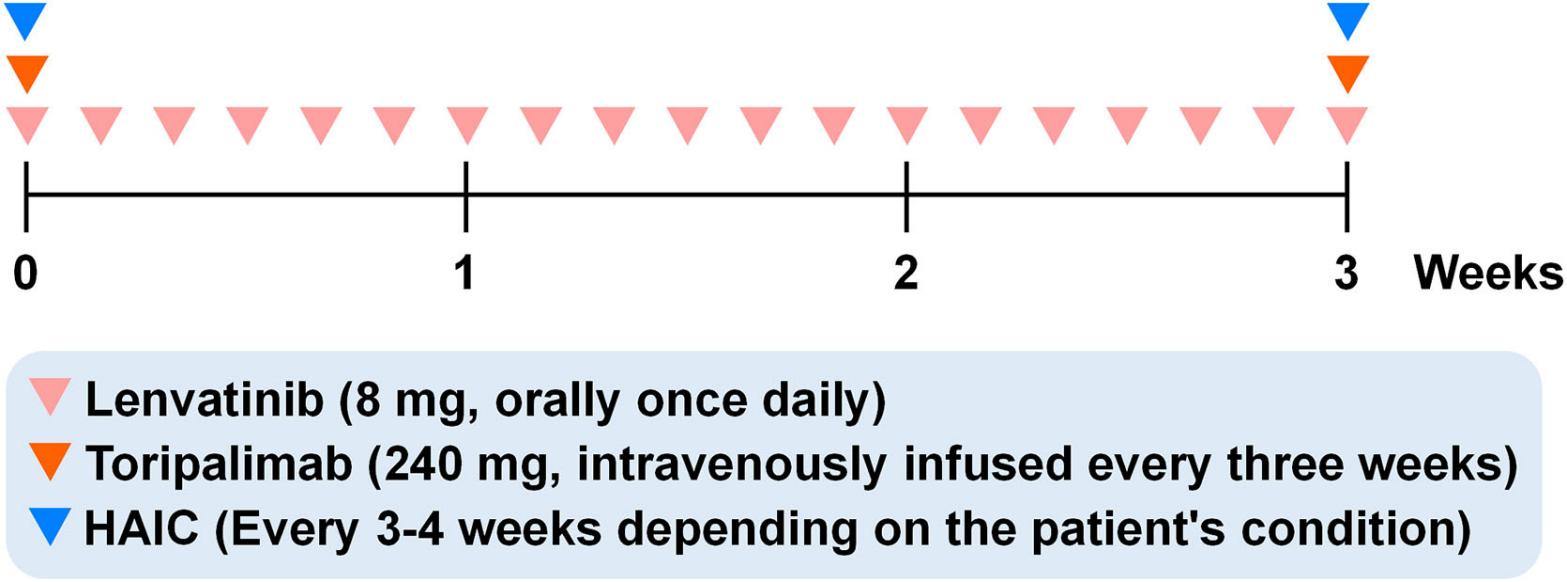
Timeline of conversion therapy regimens. It lasted from May 19, 2021 to September 11, 2021.

A multidisciplinary team (MDT) discussion concluded that the large mass with multiple satellite lesions and insufficient future liver remnant volume precluded immediate radical surgery. Based on emerging evidence for synergy between HAIC, tyrosine kinase inhibitors, and immunotherapy, the MDT consensus was to pursue an aggressive conversion therapy approach with the triple combination of lenvatinib, toripalimab, and FOLFOX4-based HAIC. The goal was not merely palliative but to achieve significant downstaging to make curative-intent resection a possibility. The treatment plan was selected based on clinical practice in 2021, guideline recommendations, drug availability, and the patient’s financial situation. Toripalimab was administered intravenously at 240 mg every 3 weeks until surgery, with premedication using dexamethasone and diphenhydramine to prevent infusion reactions. Lenvatinib was administered orally at a daily dose of 8 mg (weight-based dosing), taken in the morning with food. The patient underwent four HAIC treatments on May 19, June 11, July 2, and August 2, 2021. A microcatheter and sheath were inserted into the hepatic artery, and chemotherapy drugs were injected into the artery each time. The FOLFOX4 regimen was as follows: on the first day, oxaliplatin 130 mg and 5-fluorouracil 600 mg were pumped through catheter for 2 hours, and calcium folinate 300 mg was given intravenously. Three hundred mg of 5-fluorouracil was pumped through a catheter for 46 hours. The timeline is shown in **Figure 3**.

**Figure 4:**
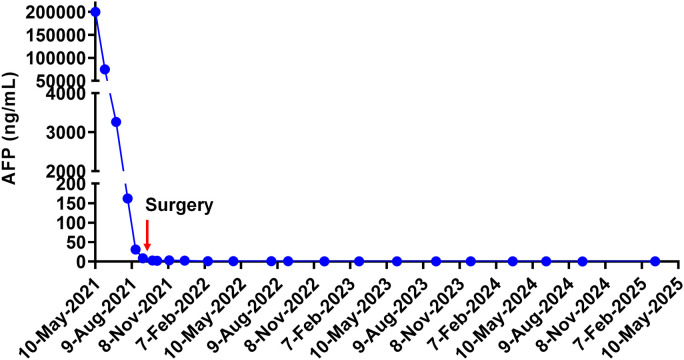
Serum alpha-fetoprotein (AFP) levels during treatment and follow-up.

The patient was monitored for adverse events every cycle according to CTCAE v5.0. Routine blood tests, liver and thyroid function, and urinalysis were performed prior to each infusion. During the treatment, the patient had only mild gastrointestinal discomfort symptoms, and the condition was improved after symptomatic treatment. In terms of biochemical indicators, only aspartate aminotransferase was slightly elevated at the beginning of treatment and returned to normal after symptomatic treatment. We were surprised to find that the volume of the liver lesion gradually decreased in the MRI. AFP also showed a significant downward trend. In September of the same year, contrast-enhanced MRI of the upper abdomen of the patient was performed again. The results showed that the mass in the right lobe of the liver was significantly smaller than before. The larger one was about 45×29×21 mm. The satellite lesions decreased, and the portal vein tumor thrombus disappeared (**Figures 1E–H**). According to the Response Evaluation Criteria in Solid Tumors 1.1 (RECIST 1.1), the disease achieved partial remission. AFP also decreased to the normal range (**Figure 4**). Therefore, after another discussion of MDT, we decided to perform right hemihepatectomy combined with inferior vena cava thrombectomy. Postoperative pathological examination showed coagulative necrosis in the tumor lesion, with increased surrounding fibrous tissue and no tumor cells at the surgical margins (**Figures 2G, H**). Given that the first two years post-surgery represent the peak period for recurrence, the patient was scheduled for re-examination every 3 months, including alternating MRI or CT scans, liver function tests, and tumor marker assays. From the third year onward, imaging was performed every 6 months, with blood tests every 3 months. The last follow-up occurred on March 13, 2025. No residual tumor was found by imaging examination after operation (**Figures 1I–L**), and AFP remained in the normal range (**Figure 4**). The patient was completely cured. The patient reported initial anxiety at diagnosis, manageable side effects during treatment, and renewed hope with the decline in AFP and tumor size. His quality of life improved significantly post-surgery, and he has returned to a normal life.

## 3 Discussion

Surgery is the primary treatment for HCC, but it is often difficult to achieve the goal of radical resection in advanced HCC. Most patients are diagnosed at intermediate or advanced stages. The remission rate of patients with BCLC stage C is only 2%-3.3%, and the median survival is only 2–3 months even if they receive sorafenib treatment (12). Intrahepatic metastasis of HCC combined with PVTT and IVCTT is very rare, but once vascular invasion occurs, the prognosis of patients is extremely poor. For some HCC with resectable intrahepatic lesions and vascular invasion, if surgical treatment is preferred, the median survival time after surgery is only 12–15 months (13). However, some unresectable advanced HCC can prolong the median survival time to 25 months after receiving non-surgical local treatment and drug therapy (14). In addition, some patients who have received a series of conversion therapies such as non-surgical local treatment and drug therapy can achieve complete remission through surgical resection.

Lenvatinib has been recommended as the first-line treatment for unresectable HCC, and its use in conversion therapy has shown promising efficacy. Studies from South Korea and the United States have confirmed the efficacy and safety of lenvatinib monotherapy in the treatment of unresectable HCC (15, 16). Tomonari et al. reported that three cases of unresectable HCC were treated with lenvatinib for 6 months and the lesions became smaller and downstaged to BCLC stage A, and then no tumor recurrence was observed during the follow-up period of 10 months after liver resection or ablation (17). Although none of these three patients had venous tumor thrombus and were diagnosed with BCLC stage B HCC, the role of lenvatinib in conversion therapy was confirmed. Chuma et al. conducted an evaluation of the efficacy of lenvatinib in patients with HCC with main portal vein tumor thrombus or liver occupying more than 50%. The results showed that the response rate of patients with tumors with main portal vein tumor thrombus was 20.0%, and the response rate of patients with tumors with more than 50% liver occupying was 29.3% (18). In addition, several reports have also shown that lenvatinib is effective for the treatment strategy of transformed hepatectomy associated with unresectable HCC (19, 20). However, the chance of radical surgical resection of unresectable HCC after receiving lenvatinib conversion regimen is only 8.4% (21), which is much lower than the expected value.

Toripalimab has shown good efficacy in a variety of cancers such as melanoma, Hodgkin’s lymphoma and nasopharyngeal carcinoma. Although there is no clinical trial of toripalimab for HCC patients, the results of a single-center retrospective study with a small sample (n=23) showed that 21.7% of HCC patients could achieve partial remission and the median progression-free survival was 48 weeks (22). The most significant advantage of toripalimab over nivolumab and pembrolizumab is its lower cost, which is less than half the cost of the first two PD-1 inhibitors for 1 year of treatment. This is one of the reasons why we chose toripalimab, taking into account the patient’s financial situation.

HAIC is not first-line in Western countries but has proven efficacy in Asian populations. The study by Iwamoto et al. showed that HAIC can prolong the overall survival (OS) of patients with locally advanced HCC compared with sorafenib (7.9 months vs 12 months) (23). A multicenter retrospective study compared the efficacy of HAIC and lenvatinib in the treatment of high tumor burden HCC. The OS of HAIC group was significantly longer than that of lenvatinib group (10.0 months vs 5.4 months), and the former helped more patients achieve better liver function (24). In addition, HAIC is significantly superior to sorafenib in terms of time to disease progression and disease control rate for HCC patients with portal vein tumor thrombus (25). Another case-control study also showed that HAIC had a better response rate and survival time than sorafenib in HCC patients with portal vein tumor thrombus (26). The specific treatment regimens of HAIC include single or combined administration, and the optimal regimen has not yet been unified. There is evidence that HAIC based on FOLFOX regimen has a good effect on advanced HCC (27). HAIC based on FOLFOX regimen can be used as an alternative treatment strategy for advanced HCC with failed transcatheter arterial chemoembolization (28).

After comprehensive evaluation of the patient’s condition, we finally selected the conversion therapy of lenvatinib, toripalimab combined with HAIC, and achieved satisfactory results. After nearly 4 months of conversion therapy, the patient’s tumor volume was significantly reduced, the portal vein tumor thrombus disappeared, and the AFP level decreased to the normal range. Even when the tumor has significantly shrunk and the portal vein tumor thrombus has disappeared, liver transplantation is a viable option. However, after complete surgical removal of the lesion, the remaining liver is sufficient to maintain the normal physiological functions of the patient. Therefore, the patient eventually underwent radical surgical resection and achieved complete remission. The decision to proceed with surgery was based on a multidisciplinary consensus weighing radiological shrinkage, biomarker normalization, and preserved liver function, rather than on a predefined, objective surgical timing parameter. During the whole treatment period, the patient had no obvious serious adverse events and complications. The liver function of this patient was Child-Pugh A before systemic therapy. Therefore, it gives us a better prerequisite for conversion therapy. However, when this patient was diagnosed with HCC, multiple metastases had occurred in the liver. The lesion was large, and accompanied by tumor thrombus in the portal vein and inferior vena cava. Therefore, conversion therapy was also extremely challenging for this patient. The exceptional outcome achieved in this case is best appreciated when contrasted with the expected prognosis for patients with similar disease burden. Patients with HCC accompanied by both PVTT and IVCTT represent one of the most challenging subgroups with a dire prognosis. Historical data indicate that without any treatment, the median overall survival (mOS) is a mere 2–4 months (4). Even with standard first-line tyrosine kinase inhibitors like sorafenib, the mOS for advanced HCC typically ranges from 10.4 to 13.9 months in clinical trials (6), and real-world studies often report lower figures. Furthermore, the rate of conversion to resection with these agents is notoriously low. For instance, a large-scale analysis reported that only 8.4% of patients initially treated with lenvatinib underwent subsequent surgical resection (21). Studies focusing on HAIC, immunotherapy and anti-angiogenic drugs have shown improved response rates, yet achieving a complete pathological response and successful resection with long-term disease-free survival, as seen in our patient, remains a relatively rare event. Among 4,930 patients with unresectable HCC from 76 studies, the objective response rate and conversion resection rate for those treated with the triple therapy were 38.52% and 30.98%, respectively (29). In this context, our patient who achieved complete radiological and biochemical response, successful curative resection with pathological confirmation of necrosis, and sustained remission beyond 40 months at the last follow-up. This represents a significant deviation from the expected outcomes and underscores the potential synergy and profound efficacy of the lenvatinib, toripalimab, and HAIC combination regimen.

The management of advanced HCC, particularly with vascular invasion, necessitates a dynamic and multimodal approach. Recent advances propose the integration of biomarker surveillance, such as AFP and des-γ-carboxy prothrombin (DCP), within structured treatment algorithms to guide therapeutic decisions and monitor response (30). In our case, serial AFP measurements provided an early and sensitive indicator of treatment response, corroborated by imaging findings. The dramatic decline in AFP from >200,000 ng/mL to within normal limits preceded and paralleled the radiological regression of tumor and thrombus, underscoring its utility in monitoring therapeutic efficacy. We propose that such biomarker kinetics, when combined with periodic imaging, can serve as a pragmatic framework for guiding treatment initiation, adjusting regimens, and determining the optimal timing for surgical intervention. Furthermore, post-resection, continued biomarker surveillance alongside imaging can facilitate early detection of recurrence, enabling prompt intervention.

However, it is regrettable that DCP was not included in the testing scope before the surgery. In the follow-up results over the past one year, we have incorporated the testing of DCP. The results show that the patient’s DCP level has remained at 20–30 mAU/mL. This is a positive sign. Furthermore, the success of treating individual cases is attributed to multiple factors. These include the patient’s underlying condition, the response to the medication, the selection of the surgical timing, and individual heterogeneity, among others. The exact efficacy and safety of this combined treatment approach in advanced liver cancer still need to be further verified in large-sample clinical trials.

## 4 Conclusion

Good liver function is one of the key factors for successful treatment. Therefore, the status of patients should be comprehensively evaluated before choosing lenvatinib, toripalimab combined with HAIC for the treatment of unresectable HCC. In addition, the timing of surgery after conversion therapy should be carefully controlled. The appropriate timing of surgery may affect the overall effect of treatment, which needs further research to explore and confirm. The successful experience reported in this case shows that lenvatinib, toripalimab combined with HAIC have a good prospect as a conversion therapy for HCC with intrahepatic metastasis and multiple venous tumor thrombi. However, it is necessary to further validate this protocol in large sample clinical studies.

## Data Availability

The original contributions presented in the study are included in the article/supplementary material. Further inquiries can be directed to the corresponding author.
